# Spectral Changes of EEG Following a 6-Week Low-Dose Oral Ketamine Treatment in Adults With Major Depressive Disorder and Chronic Suicidality

**DOI:** 10.1093/ijnp/pyad006

**Published:** 2023-02-15

**Authors:** T E Anijärv, A T Can, C C Gallay, G A Forsyth, M Dutton, J S Mitchell, D F Hermens, J Lagopoulos

**Affiliations:** Thompson Institute, University of the Sunshine Coast, Birtinya, Queensland, Australia; Thompson Institute, University of the Sunshine Coast, Birtinya, Queensland, Australia; Thompson Institute, University of the Sunshine Coast, Birtinya, Queensland, Australia; Thompson Institute, University of the Sunshine Coast, Birtinya, Queensland, Australia; Thompson Institute, University of the Sunshine Coast, Birtinya, Queensland, Australia; Thompson Institute, University of the Sunshine Coast, Birtinya, Queensland, Australia; Thompson Institute, University of the Sunshine Coast, Birtinya, Queensland, Australia; Thompson Institute, University of the Sunshine Coast, Birtinya, Queensland, Australia

**Keywords:** EEG, spectral analysis, ketamine, suicidality, depression

## Abstract

**Background:**

Ketamine has considerable therapeutic potential in alleviating major depressive disorder and chronic suicidality. However, the clinical diagnosis of neuropsychiatric disorders requires more robust diagnostic criteria. Electroencephalography (EEG) has shown promise in classifying depressive and suicidal patients from healthy individuals. The present study aimed to identify changes in the spectral properties of EEG in patients with major depressive disorder and chronic suicidality after completing the 6-week Oral Ketamine Trial on Suicidality with follow-up occurring 4 weeks after final ketamine treatment and determine associations between EEG spectral output and clinical symptoms.

**Methods:**

Participants (n = 25) had 4-minute eyes closed resting state EEG recorded at frontal, temporal, centro-parietal, and occipital regions. Spectral analysis was performed with Welch’s power spectrum density method, and the power of 4 distinct frequency bands was analyzed: theta, alpha, low-beta, and high-beta. Correlation analyses between changes in clinical symptoms and spectral power were conducted using Spearman’s ranked correlation.

**Results:**

Between pre- and posttreatment, only centro-parietal alpha power decreased. Between posttreatment and follow-up, centro-parietal alpha increased again in addition to increases in temporal alpha, centro-parietal and temporal theta, and occipital low-beta and decreases in occipital theta and temporal low-beta. Additionally, the decrease of occipital theta positively correlated with clinical subscales for depression and stress.

**Conclusions:**

EEG spectral analysis revealed significant changes in theta, alpha, and low-beta frequency bands. Alpha band showed initial changes after treatment; however, this trended back toward baseline levels after the treatment cessation. In contrast, theta and low-beta showed significant power changes only after the treatment had ended.

**Significance Statement**

Our study is the first, to our knowledge, to examine electrophysiological changes in adults with major depression and chronic suicidality following a low-dose ketamine treatment with oral administration. While previous studies have confirmed the feasibility and tolerability of oral ketamine as a treatment for chronic suicidality, the present study reports significant changes in the spectral properties of EEG. Alpha band power displayed changes throughout the trial, during the active treatment phase, and after the treatment had ended. Theta and low-beta band powers showed changes only after the treatment period had ended, between posttreatment and later follow-up. Theta band also correlated the most with clinical symptoms.

## INTRODUCTION

Major depressive disorder (MDD) is one of the most prevalent and disabling psychiatric disorders globally, affecting 1 in 6 adults in their lifetime (Otte et al., 2016). Besides the significant burden conferred by MDD alone, it is a common co-morbidity to physical and mental disorders, and, importantly, it is associated with an increased risk of suicide. Clinically, suicidality is characterized by recurrent and intrusive contemplations, wishes, and preoccupations with death and suicide. Annually, more than 700 000 deaths in the world are attributed to suicide (World Health Organization, 2021), of which the high prevalence of MDD substantially contributes to the 10.6 and 7.2 years of life lost in men and women, respectively (Chesney et al., 2014). Currently, the clinical diagnosis of MDD and suicidality rely on self-report measures in combination with clinical screening. To establish more robust diagnostic criteria that can address the significant variability in disorder pathogenesis and presentation, considerable research effort has been focused on finding biomarkers for neuropsychiatric disorders such as MDD and suicidality (for reviews, see de Aguiar Neto and Rosa, 2019; Newson and Thiagarajan, 2019; Dev et al., 2022).

The spatio-temporal profile of neural circuit dynamics, and information processing within the brain, is predominantly defined by the dynamic balance of excitatory and inhibitory transmission of neurons (Sohal and Rubenstein, 2019). An emergent property of excitatory and inhibitory neural transmission that lends itself to measurement is the synchronized activity that arises from populations of neurons (Jackson and Bolger, 2014). Electroencephalography (EEG) is a non-invasive tool frequently used to measure these brain activity signals (Biasiucci et al., 2019), which, owing to technological advancements, has garnered renewed interest as a valuable method for determining biomarkers for depression and drug response (de Aguiar Neto and Rosa, 2019; Ip et al., 2021a, 2021b). In recent clinical studies, EEG has shown promise in classifying depressive and suicidal patients from healthy individuals (Grin-Yatsenko et al., 2010; Jaworska et al., 2012; Lee et al., 2017) and in assessing ketamine as a treatment for depression (Cao et al., 2019; McMillan et al., 2020; de la Salle et al., 2022).

Ketamine, an NMDA receptor noncompetitive antagonist, has shown considerable therapeutic potential in alleviating MDD and suicidality at subanesthetic, low doses (0.5–3.0mg/kg; Can et al., 2021; Gallay et al., 2021). Mechanistically, these effects are associated with NMDA receptor–mediated changes in excitatory and inhibitory neurotransmission (i.e., glutamatergic and GABAergic activity, respectively) in the medial prefrontal cortex, anterior cingulate cortex, and hippocampus (Shinohara et al., 2021), driving a period of plasticity-induced structural and functional remodeling (Wong et al., 2016).

One of the most common techniques to acquire information from EEG data is to transform the signals from time domain to frequency domain and describe the signals using well-known frequency bands: delta, theta, alpha, beta, and gamma (de Aguiar Neto and Rosa, 2019). However, delta and gamma bands can be artifactual (Muthukumaraswamy, 2013; Castermans et al., 2014). This method, termed band power or spectral analysis, of both global and region-specific EEG signals formed from cortical and subcortical circuits has yielded some valuable insights into the effect of ketamine on neural circuit dynamics. A study conducted by Grin-Yatsenko et al. (2010) compared EEG power spectra between 111 depressed and 526 healthy patients, reporting increased power in theta, alpha, and beta bands at parietal and occipital regions for the depressed group. Moreover, alpha band power has been found to be greater in frontal and parietal regions for unmedicated MDD patients compared with healthy controls (Jaworska et al., 2012). In addition, increased relative theta band power has been found in healthy individuals with higher suicidality in frontal and central regions (Lee et al., 2017). Abnormally higher theta, alpha, and beta band powers could display an imbalance of excitatory and inhibitory transmission in neural circuits, which thereby leads to a psychiatric state or disorder. A randomized double-blind trial including participants with treatment-resistant MDD found that i.v. subanaesthetic dosage (0.5 mg/kg) of ketamine decreased power in theta, alpha (parietal region), and beta (centro-parietal region) bands immediately post-infusion while decreasing depressive symptoms and suicidality. In contrast, 240 minutes after the infusion, the power increased in all the bands toward the baseline values (de la Salle et al., 2022). Another study by Cao et al. (2019) found alpha band power to be increased at frontal regions 240–250 minutes after subanaesthetic ketamine infusion for participants with treatment-resistant depression. In a recent randomized, double-blind, active placebo-controlled crossover trial, resting-state EEG was recorded during infusion of ketamine (subanesthetic dose) or active placebo (remifentanil) in 30 participants with MDD, and the observed spectral changes included an increase in theta and decreases in delta, alpha, and beta band powers; however, none of these changes predicted treatment response (McMillan et al., 2020).

This present study aimed to identify changes in the spectral properties of EEG in patients with chronic suicidality (and diagnosed with MDD) after a 6-week low-dose oral ketamine treatment and determine if there was an association between EEG spectral output and clinical symptoms. Considering previous findings (Grin-Yatsenko et al., 2010; Jaworska et al., 2012; Lee et al., 2017; Cao et al., 2019; McMillan et al., 2020; de la Salle et al., 2022), it was hypothesized that compared with pretreatment, EEG patterns at the posttreatment (6-week) timepoint would show a significant (1) decrease of theta band power in all regions (frontal, temporal, centro-parietal, occipital); (2) decrease of alpha band power in the centro-parietal region; and (3) decrease of low- and/or high-beta band power in the centro-parietal region. Four weeks after the final ketamine treatment (i.e., follow-up at 10-week), it was predicted that the direction of changes in EEG power spectra would trend back toward the pretreatment values (i.e., increase of power in the aforementioned bands at the same regions) and the pattern of clinical symptoms would follow suit.

## Method

### Clinical Trial

**Figure 1.**
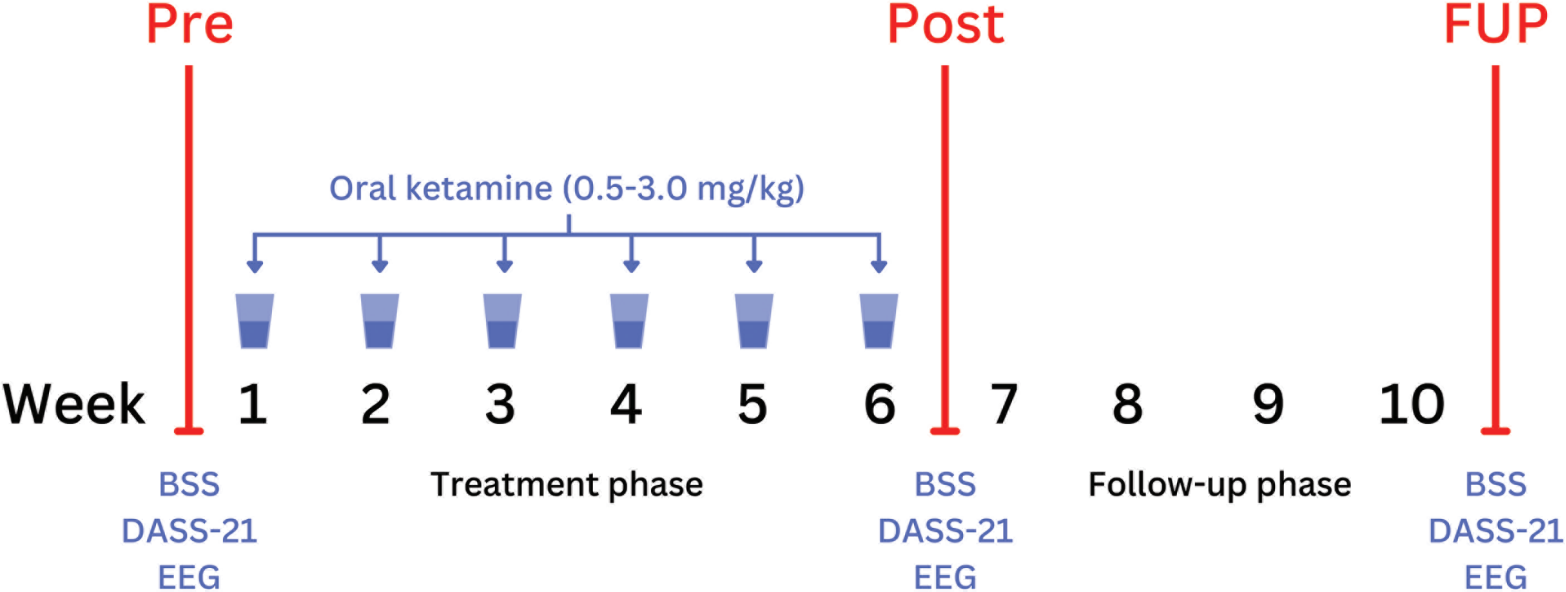
Oral Ketamine Trial on Suicidality (OKTOS) design.

This study formed part of a larger open-label clinical trial, Oral Ketamine Trial on Suicidality (OKTOS), conducted at the Thompson Institute between August 2018 and November 2019 (Can et al., 2021). The intervention consisted of 6 weeks of flexible-dose (titration from 0.5 up to 3.0 mg/kg) treatment with oral ketamine and a 4-week follow-up phase without the treatment (nil ketamine). All participants received a total of 6 oral, subanesthetic doses of ketamine for 6 weeks, 1 dose per week. The trial had 3 major timepoints (Figure 1): (1) pretreatment (Pre; i.e., up to 14 days prior to first treatment); (2) posttreatment (Post; i.e., 4–7 days after final ketamine treatment); and (3) follow-up (FUP; i.e., 28–32 days after final ketamine treatment).

The trial included a broad range of clinical, neurophysiological, neuroimaging, and biochemical measures (i.e., MRI, EEG, blood tests, urinalysis, clinical scales). This study utilized data only from EEG recordings and 2 clinical scales. The primary clinical outcome measure was reduction in suicidality with ketamine treatment, and this was determined by Beck Scale for Suicide Ideation (BSS) (Can et al., 2021). Secondary clinical measures used in this study included the Depression Anxiety Stress Scale 21 (DASS-21) (Beaudequin et al., 2020; Dutton et al., 2022). In total, assessments at each major timepoint required approximately 8 hours. The clinical scales and biophysical data (i.e., blood tests, urinalysis) were the first items to be collected, and remaining measures were acquired based on the availability of shared resources and were therefore not collected in any specific order. For this reason, EEG data acquisition occurred at variable times during the day between 8:00 am and 6:00 pm. Participants were offered fuel vouchers to assist in the travel to their assessments and weekly treatments at the Thompson Institute if requested.

Ethics approval was obtained through the Bellberry Limited Ethics Committee (2017-12-982) and ratified by the University of the Sunshine Coast Human Research Ethics Committee (A181101). All study activities were conducted in accordance with the Australian National Statement on Ethical Conduct in Human Research (2007) and conformed with the Declaration of Helsinki ethical principles for medical research involving human subjects. This study was registered on the Australian Clinical Trials Registry ( https://www.australianclinicaltrials.gov.au/anzctr/trial/ACTRN12618001412224).

The anonymized data that support the findings of this study are available from the corresponding author upon reasonable request.

### Participants

Of the 32 participants who completed the trial, 25 were included in the current study due to participant withdrawal (n = 2 after week 6) or missing electrophysiological recording session(s) (n = 1 for Pre, n = 1 for Post, n = 3 for FUP). These 25 participants included 11 males and 14 females aged 22 to 71 years (mean = 46.41 years, SD = 14.12). The participants were suffering from chronic suicidality and were referred to the trial by their local general practitioners. All participants had a diagnosis of MDD (DSM-V), and 76% had comorbid mental disorders, 92% reported ongoing use of psychotropic medications (see Can et al., 2021 for details).

### Electrophysiological Recordings

Participants were seated in a quiet, dimly lit room. Four minutes of eyes closed resting-state EEG data were acquired according to standard pharmaco-EEG procedures (Jobert et al., 2012) with BioSemi ActiveTwo 32-channel system (Biosemi B.V, Amsterdam, the Netherlands) at a sampling rate of 1024 Hz. Scalp electrodes (Ag/AgCl active electrodes impedances <40 kΩ) were localized according to the international 10/20 layout. Six additional electrodes were placed, including 2 (left and right) mastoids and 4 electrooculographic (EOG) channels for obtaining horizontal and vertical eye movements.

### Electroencephalographic Analysis

An in-house EEG data processing pipeline, EEG-pyline, was used for signal preprocessing and spectral analysis efforts (Anijärv, 2022). The specific code (i.e., Jupyter notebook) used for this study can be found in the ‘studies’ folder within the GitHub repository.

#### Signal Preprocessing

**—**All signals were filtered with a 0.5- to 30-Hz band-pass filter (FIR with Hamming window, 1-pass, 0-phase, non-causal). EOG artefacts, including eye movements, were removed by computing signal-space projection vectors using specific EOG channels acquired during EEG recording and applying these projections to the EEG signal to remove the artefacts. The previously mentioned steps were conducted using the MNE package for Python (Gramfort et al., 2013). The time series was divided into equal-sized, consecutive, 5-second epochs without any overlap between epochs. Epochs were cleaned from artefacts in all channels using the Python package Autoreject (Jas et al., 2016, 2017). The resulting EEG signals for each participant were visually screened using global field power plots to check whether the magnitude of a signal was in similar scale throughout the whole signal, thus ensuring the removal of large artefacts.

#### Spectral Analysis

**—**The processed EEG signals were transformed into the frequency domain by estimating power spectrum density (PSD) using Welch’s method (Welch, 1967), with a 2-second Hamming window (50% overlap). Average PSD values (µV^2^/Hz) were calculated for 4 frequency bands: theta (4–7.9 Hz), alpha (8–12 Hz), low-beta (12.1–18 Hz), and high-beta (18.1–30 Hz). All 32 channels were averaged into 4 brain regions: frontal (Fp1/2, AF3/4, F3/4, F7/8, Fz), temporal (FC5/6, T7/8, CP5/6, P7/8), centro-parietal (FC1/2, C3/4, Cz, CP1/2, P3/4, Pz), and occipital (PO3/4, O1/2, Oz). Additionally, signal reliability was checked for each band by calculating z-scores using median and absolute median deviation across all the epochs to determine if the PSD values fluctuated across time. As the participants were recorded in resting state, the power spectra were expected to not change more than 2 absolute median deviations across epochs. Delta band (1–3.9 Hz) did not pass this reliability test within most of the participants and therefore was not included in further analysis.

#### Correlation With Clinical Outcomes

**—**Correlation coefficients for changes between timepoints (i.e., Pre, Post, and FUP) on 2 self-reported measures, the BSS and DASS-21, and EEG power at all 4 frequency bands were calculated. DASS-21 includes 3 different subscales: DASS-D for depression, DASS-A for anxiety, and DASS-S for stress. For calculating the coefficients, Spearman correlation was used due to the nonparametric distribution of the data (Mukaka, 2012). Thresholds for the coefficients were set as the following magnitudes: ± 0.3–0.5 for low correlation, ± 0.5–0.7 for moderate correlation, and ± 0.7–1.0 for high correlation.

### Statistics

To evaluate EEG power differences across different timepoints (i.e., Pre, Post, and FUP), a Wilcoxon signed-rank test was used. The nonparametric test was required since the EEG data did not meet the parametric assumptions (i.e., not normally distributed), and participants were compared with themselves across timepoints (i.e., paired samples). For statistical testing of the Spearman correlation coefficients, 2-tailed *t* test distributions were used, with significance set at *P *< .05. The statistical analysis and data visualization were performed with the support of Pandas (McKinney, 2010; Reback et al., 2022), NumPy (Harris et al., 2020), SciPy (Virtanen et al., 2020), Matplotlib (Hunter, 2007), and Seaborn (Waskom, 2021) packages for Python. The EEG data are summarized and presented by measures of central tendency and dispersion, both by median (M) with interquartile range (IQR) and mean with SD (Pupovac and Petrovečki, 2011).

## RESULTS

### Spectral Analysis

#### Theta Band

**Figure 2.**
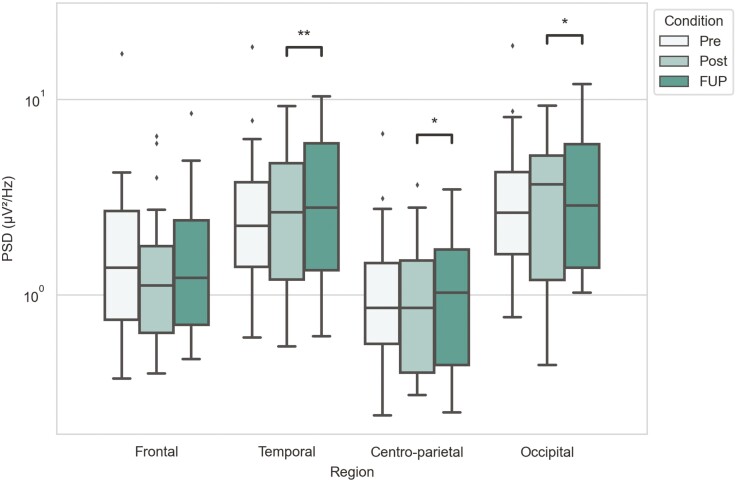
Theta band power for different timepoints and brain regions. Power spectra values have been plotted in a logarithmic scale y-axis. Statistical significance annotations are as follows: *P < .05, **P < .01; exact *P* values can be found in supplementary Table 1.1.

**—**Theta band displayed significant changes only between the posttreatment and follow-up timepoints. There were increases in power between posttreatment and follow-up timepoints at the temporal (M_Post_ = 2.641, IQR_Post_ = [1.194; 4.694] → M_FUP_ = 2.784, IQR_FUP_ = [1.334; 5.932]; *P*_Post- FUP_ = 0.006) and centro-parietal (M_Post_ = 0.857, IQR_Post_ = [0.399; 1.492] → M_FUP_ = 1.025, IQR_FUP_ = [0.437; 1.698]; *P*_Post-FUP_ = 0.019) regions and a decrease in power for the occipital region (M_Post_ = 3.670, IQR_Post_ = [1.189; 5.140] → M_FUP_ = 2.854, IQR_FUP_ = [1.375; 5.874]; *P*_Post-FUP_ = 0.012) (Figure 2; supplementary Table 1.1).

#### Alpha Band

**Figure 3.**
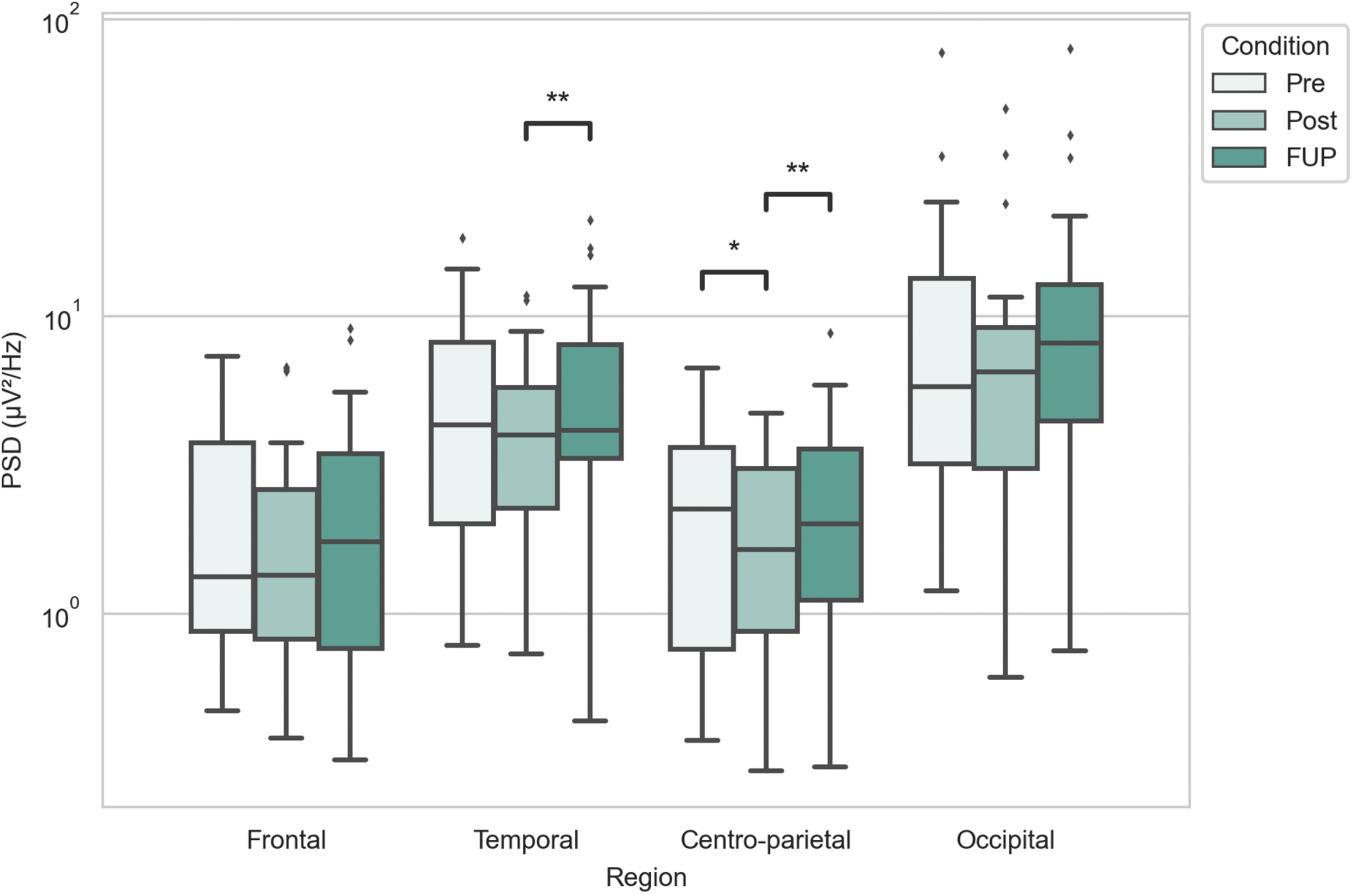
Alpha band power for different timepoints and brain regions. Power spectra values have been plotted in a logarithmic scale y-axis. Statistical significance annotations are as follows: *P < .05, **P < .01; exact *P* values can be found in supplementary Table 1.2.

**—**Alpha band power at centro-parietal region decreased between pretreatment and posttreatment (M_Pre_ = 2.248, IQR_Pre_ = [0.758; 3.605] → M_Post_ = 1.635, IQR_Post_ = [0.871; 3.077]; *P*_Pre-Post_ = 0.048), followed by an increase in power between posttreatment and follow-up timepoints (M_Post_ = 1.635, IQR_Post_ = [0.871; 3.077] → M_FUP_ = 2.004, IQR_FUP_ = [1.109; 3.574]; *P*_Post-FUP_ = 0.007). Analyses similarly revealed an increase in power between posttreatment and follow-up timepoints at the temporal region (M_Post_ = 3.989, IQR_Post_ = [2.263; 5.742] → M_FUP_ = 4.130, IQR_FUP_ = [3.321; 8.034]; *P*_Post_-_FUP_ = 0.003) (Figure 3; supplementary Table 1.2).

#### Low-Beta Band

**Figure 4.**
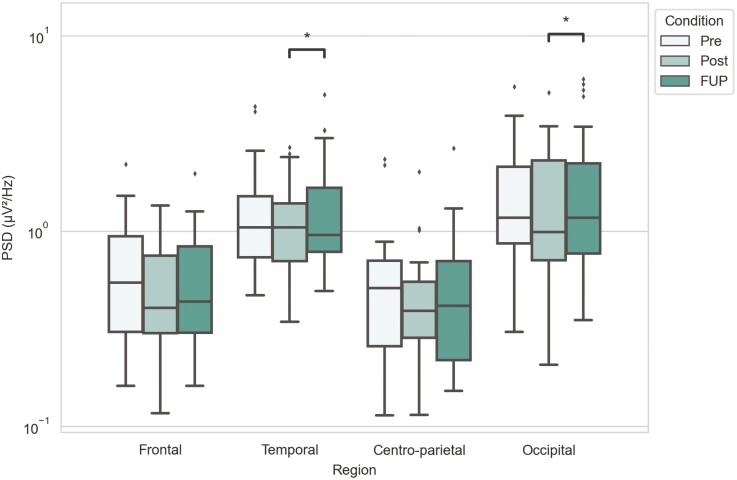
Low-beta band power for different timepoints and brain regions. Power spectra values have been plotted in a logarithmic scale y-axis. Statistical significance annotations are as follows: *P < .05; exact *P* values can be found in supplementary Table 1.3.

**—**Low-beta band showed an increase in power between posttreatment and follow-up timepoints at the occipital region (M_Post_ = 0.987, IQR_Post_ = [0.708; 2.297] → M_FUP_ = 1.170, IQR_FUP_ = [0.767; 2.224]; *P*_Post-FUP_ = 0.027). Also, the power decreased between posttreatment and follow-up at the temporal region (M_Post_ = 1.042, IQR_Post_ = [0.701; 1.388] → M_FUP_ = 0.953, IQR_FUP_ = [0.781; 1.665]; *P*_Post-FUP_ = 0.042) (Figure 4; supplementary Table 1.3).

#### High-Beta Band

**Figure 5.**
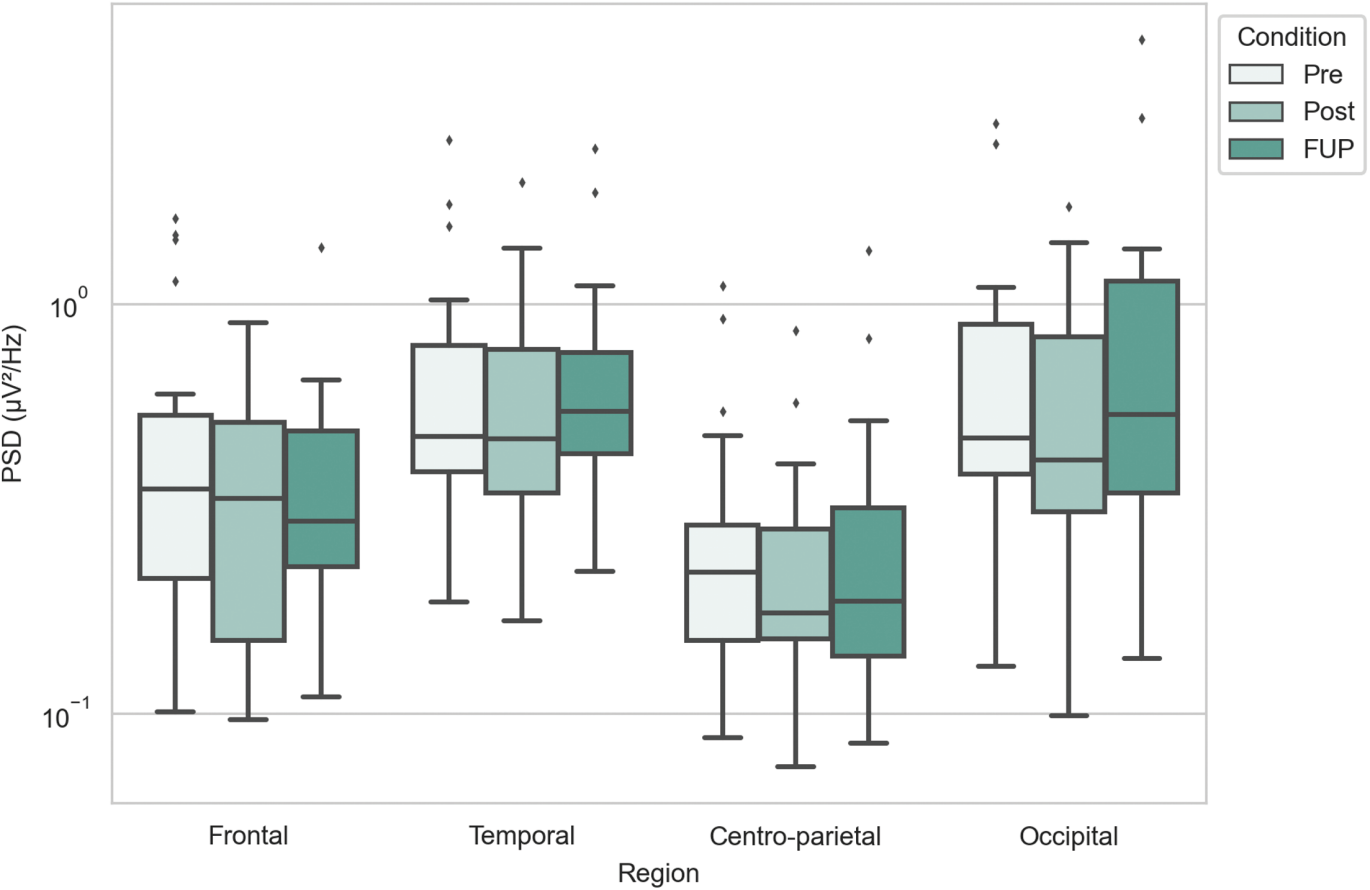
High-beta band power for different timepoints and brain regions. Power spectra values have been plotted in a logarithmic scale y-axis. Statistical significance levels, that is, *P* values, can be found in supplementary Table 1.4.

**—**When comparing high-beta band power between pretreatment and posttreatment, posttreatment and follow-up, or pretreatment and follow-up, no statistically significant differences were found (Figure 5; supplementary Table 1.4).

### Correlation Between Power Spectra and Clinical Outcomes

**Figure 6.**
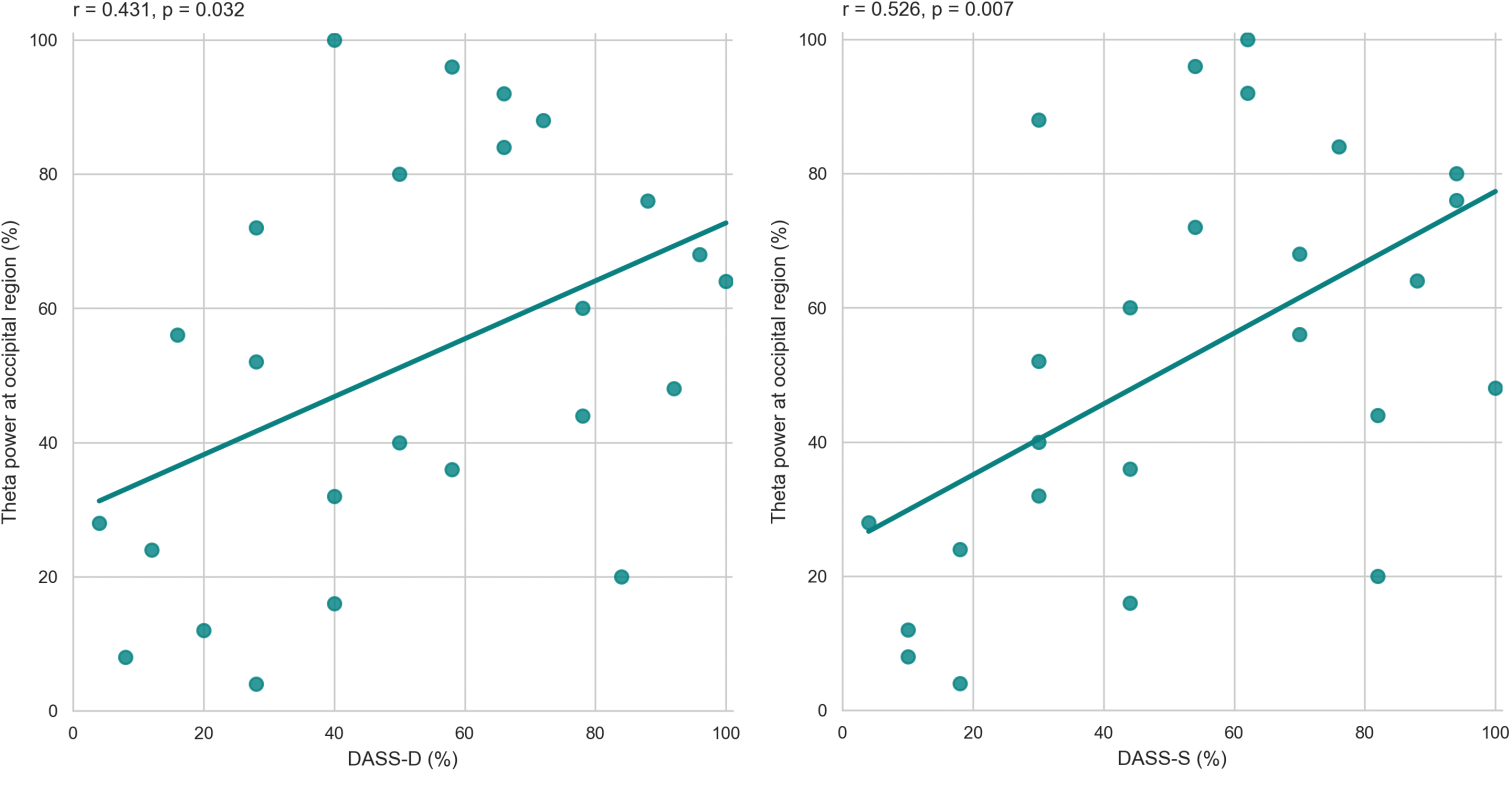
Theta at the occipital region between posttreatment and follow-up timepoints (also had significant change in power; see Figure 1) positively correlates with Depression Anxiety Stress Scale (DASS-S) (left) and for depression (DASS-D) (right). Displayed data are ranked and plotted in percentiles.

None of the power spectra changes within the 4 bands correlated with change in BSS among the 3 timepoint pairs (i.e., Pre-Post, Post-FUP, Pre-FUP). However, the secondary clinical outcome measure, DASS-21, showed several correlations with the EEG spectral data. Change in DASS-D correlated with Post-FUP change in occipital theta (r = 0.431, *P* = .032; Figure 6), frontal low-beta (r = −0.464, *P* = .019), and frontal high-beta (r = −0.571, *P* = .003) and with Pre-FUP change in occipital low-beta (r = −0.421, *P* = .036), temporal high-beta (r = −0.504, *P* = .010), and centro-parietal high-beta (r = −0.420, *P* = .036).

Change in DASS-A correlated with Pre-Post changes in occipital theta (r = −0.522, *P* = .007) and with Pre-FUP changes in centro-parietal theta (r = −0.404, *P* = .045), temporal alpha (r = −0.455, *P* = .022), and temporal high-beta (r = −0.443, *P* = .027).

Change in DASS-S correlated with Pre-Post changes in temporal theta (r = −0.585, *P* = .002), occipital low-beta (r = −0.506, *P* = .010), occipital theta (r = −0.659, *P* = .0003), and temporal low-beta (r = −0.528, *P* = .007); with Post-FUP changes in occipital theta (r = 0.526, *P* = .007; Figure 6); and with Pre-FUP changes in temporal alpha (r = −0.402, *P* = .046), temporal high-beta (r = −0.595, *P* = .002), and occipital high-beta (r = −0.399, *P* = .048) (supplementary Tables 2.1, 2.2, 2.3, 2.4).

## DISCUSSION

The main objective of this study was to explore spectral changes of EEG in patients with MDD and chronic suicidality following 6 weeks of low-dose oral ketamine treatment. A second objective was to investigate correlations between changes in EEG spectral results and changes in clinical measures of suicidality, depression, anxiety, and stress.

**Figure 7.**
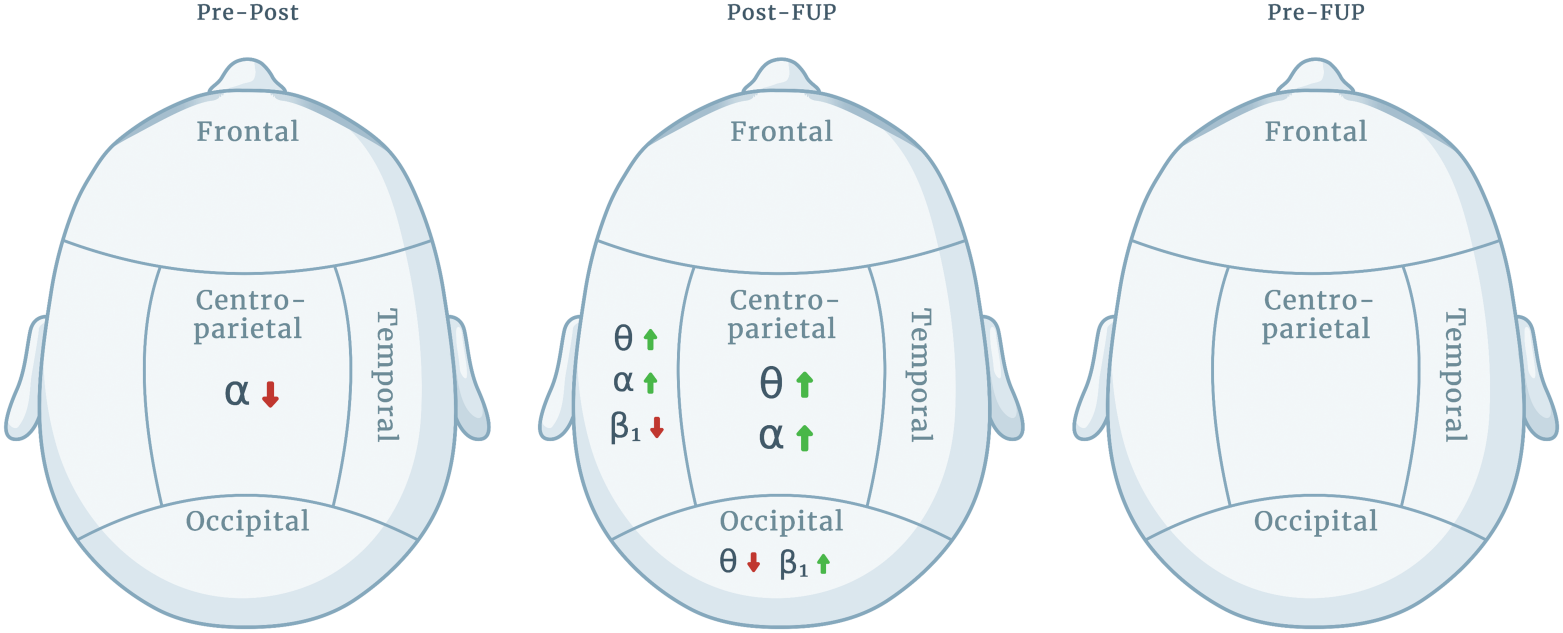
Illustrative visualization of brain regions and significant changes in band powers between Pre-Post (left), Post-FUP (middle), and Pre-FUP (right) timepoints. Created with BioRender.com.

The most conclusive finding was the hypothesized alpha band power change at the centro-parietal region. Between pretreatment and posttreatment timepoints, the alpha band power decreased at the centro-parietal region. Following the final ketamine treatment between posttreatment and follow-up, alpha band power increased again at the same region (Figures 3 and 7). Similar changes were also reported in recent study investigating low-dose ketamine as treatment for depression (McMillan et al., 2020; de la Salle et al., 2022). Additionally, our results showed alpha band power significantly increased between the posttreatment and follow-up timepoints at the temporal region. However, at that same region, a decrease in power between pretreatment and posttreatment was not statistically significant. As alpha band power has been found to be higher in depressive patients compared with healthy controls (Grin-Yatsenko et al., 2010; Jaworska et al., 2012), our findings suggest that low-dose oral ketamine decreases alpha band power at the centro-parietal region, but the changes revert close to pretreatment levels after ketamine treatment has ceased. We did not find any statistically significant change between alpha band power between pretreatment and follow-up (Figure 3), which suggests the effects of ketamine are transient in terms of changes in alpha activity.

For theta band, we did not find any power changes immediately following the treatment period (i.e., posttreatment), but there were significant changes between posttreatment and follow-up timepoints; power was decreased at the occipital region and increased at the temporal and centro-parietal regions (Figures 2 and 7). Interestingly, de la Salle et al. (2022) reported an increase in theta band power following final ketamine treatment. However, these results are not directly comparable, as changes were measured 2 hours posttreatment compared with 28–32 days following final ketamine treatment in OKTOS. Other clinical studies (which did not include ketamine treatment) have found theta power to be higher in participants with depression compared with healthy controls. For example, Grin-Yatsenko et al. (2010) found theta power to be increased at parietal and occipital regions, while Lee et al. (2017) reported this at the frontal and central regions. Based on these studies, the occipital theta decrease observed here could represent a delayed positive treatment effect due to ketamine-induced neuroplastic changes. In contrast, increase in temporal and centro-parietal theta power would suggest the opposite—that treatment effects have ceased, and the depressive pathophysiology returns. Thereby, theta band power changes in our study are inconclusive, and more studies are needed to confirm the function of theta band power in depression and suicidality and whether this is ameliorated by ketamine treatment.

High-beta band showed no significant power changes across timepoints (Figure 5), but low-beta power between posttreatment and follow-up decreased at the temporal region and increased at the occipital region (Figures 4 and 7). Our findings for beta bands were not consistent with previous findings by de la Salle et al. (2022), potentially due to the timing of measurements at posttreatment and follow-up. In terms of cognitive functions, a recent study found that low-beta (and alpha) band powers at the parieto-occipital region reflect one’s sense of agency (Bu-Omer et al., 2021), whereas a distorted sense of agency has been suggested to be linked to several neuropsychiatric disorders, including depression (Haggard, 2017). Beta band activity was also previously related to anxiety (Freeman and Quian Quiroga, 2013; Abhang et al., 2016), which itself has been used to predict suicidal ideation (Bentley et al., 2016), indicating an indirect relationship. Therefore, the ketamine therapeutic effect on depression and suicidality reported here could be mediated by changes in beta band activity that influence sense of agency and/or symptoms of anxiety, but further studies are needed for more precise conclusions.

While none of the power spectra changes correlated with the main clinical outcome (BSS), several significant relationships with the secondary measure (DASS-21) were found. One of the most relevant correlation findings was that the theta band power at the occipital region showed a moderate positive correlation with DASS-S and weak positive correlation with DASS-D between posttreatment and follow-up timepoints (Figure 6). This is relevant because the changes at the occipital region for theta band power between posttreatment and follow-up were also statistically significant (Figure 2). There exists a paucity of research examining long-lasting electrophysiological effects of ketamine in the suicidal population. For this reason, the relationship between ketamine’s effect on both clinical and EEG measures remains unclear, but future studies with a larger sample size and randomized control study design should examine the utility of EEG as a biomarker in predicting long-term antidepressant response to ketamine. Additionally, the lack of relationship between EEG spectral changes and clinical symptoms could be due to low-frequency EEG oscillations having a stronger correlation with psychotomimetic effects rather than antidepressant effects of ketamine (Nottage et al., 2022).

In this study, there were several limitations that may have impacted the results. Firstly, the sample size was relatively small, which may have impacted the results. Secondly, this study focused only on the whole group-level ketamine treatment effects without categorically examining the differences between (1) responders (n = 17; 68%) and nonresponders (n = 8; 32%), according to BSS changes from Pre to Post timepoints; and (2) prolonged responders (n = 11; 44%) and prolonged nonresponders (n = 14; 56%), according to BSS changes from Pre to FUP timepoints. According to Can et al. (2021), “response” or “prolonged response” was defined by a >50% improvement in BSS score from Pre to Post/FUP timepoints or BSS score <6 in Post/FUP timepoint. Thirdly, the neurological effects of concurrent medications could not be excluded as most of the participants were taking various psychotropic medications throughout the trial. Fourthly, this study focused on chronic suicidality (i.e., patients with suicidality for at least 6 months), and there may be different outcomes if this period of duration changes. Finally, the current study design did not include healthy controls, placebo, or active comparator groups for comparison, which would be necessary to draw more robust conclusions regarding the spectral changes of EEG.

This study found that long-term, low-dose oral ketamine treatment caused significant changes in EEG power spectra in adults with MDD and chronic suicidality. However, further studies of low-dose oral ketamine in suicidality are needed using a randomized control study design and larger sample size to address the limitations of the current open-label trial.

## Supplementary Materials

Supplementary data are available at *International Journal of Neuropsychopharmacology (IJNPPY)* online.

pyad006_suppl_Supplementary_Material
