# Supplementary material for: Spectral Changes of EEG Following a 6-Week Low-Dose Oral Ketamine Treatment in Adults With Major Depressive Disorder and Chronic Suicidality
Source: Int J Neuropsychopharmacol. 2023 Feb 15;26(4):259–67. doi: 10.1093/ijnp/pyad006 (PMC10109122; doi:10.1093/ijnp/pyad006)
Supplement: pyad006_suppl_Supplementary_Material [file pyad006_suppl_supplementary_material.docx]

# Supplementary material

**Supplementary Table S1.1.** Theta power at different timepoints and brain regions with comparisons (* p<.05; ** p<.01).

|  | Pre-treatment (Pre) | | Post-treatment (Post) | | Follow-up (FUP) | | Comparison | | | |
| --- | --- | --- | --- | --- | --- | --- | --- | --- | --- | --- |
|  | Mean (SD) | Median (IQR) | Mean (SD) | Median (IQR) | Mean (SD) | Median (IQR) | p_Pre-Post_ | | p_Post-FUP_ | p_Pre-FUP_ |
| Frontal | 2.227 (3.251) | 1.375 (0.743- 2.677) | 1.680 (1.590) | 1.111 (0.638- 1.769) | 1.814 (1.782) | 1.218 (0.699- 2.397) | 0.220 | 0.287 | | 0.396 |
| Temporal | 3.453 (3.698) | 2.252 (1.387- 3.762) | 3.018 (2.173) | 2.641 (1.194- 4.694) | 3.763 (2.939) | 2.784 (1.334- 5.932) | 0.634 | 0.006** | | 0.090 |
| Centro-parietal | 1.286 (1.353) | 0.853 (0.559- 1.449) | 1.145 (0.939) | 0.857 (0.399- 1.492) | 1.268 (1.006) | 1.025 (0.437- 1.698) | 0.751 | 0.019* | | 0.508 |
| Occipital | 3.899 (3.986) | 2.630 (1.612- 4.236) | 3.410 (2.525) | 3.670 (1.189- 5.140) | 3.986 (3.136) | 2.854 (1.375- 5.874) | 0.525 | 0.012* | | 0.312 |

**Supplementary Table S1.2.** Alpha power at different timepoints and brain regions with comparisons (* p<.05; ** p<.01).

|  | Pre-treatment (Pre) | | Post-treatment (Post) | | Follow-up (FUP) | | Comparison | | | |
| --- | --- | --- | --- | --- | --- | --- | --- | --- | --- | --- |
|  | Mean (SD) | Median (IQR) | Mean (SD) | Median (IQR) | Mean (SD) | Median (IQR) | p_Pre-Post_ | | p_Post-FUP_ | p_Pre-FUP_ |
| Frontal | 2.556 (2.193) | 1.328 (0.867- 3.755) | 2.107 (1.964) | 1.343 (0.820- 2.613) | 2.543 (2.343) | 1.738 (0.759- 3.439) | 0.381 | 0.200 | | 0.596 |
| Temporal | 5.513 (4.505) | 4.302 (1.999- 8.163) | 4.408 (2.952) | 3.989 (2.263- 5.742) | 6.288 (5.264) | 4.130 (3.321- 8.034) | 0.173 | 0.003** | | 0.113 |
| Centro-parietal | 2.509 (2.017) | 2.248 (0.758- 3.605) | 1.937 (1.361) | 1.635 (0.871- 3.077) | 2.522 (2.068) | 2.004 (1.109- 3.574) | 0.048* | 0.007** | | 0.634 |
| Occipital | 11.417 (15.845) | 5.802 (3.177- 13.386) | 9.316 (11.217) | 6.499 (3.066- 9.136) | 12.914 (16.872) | 8.094 (4.424- 12.789) | 0.411 | 0.113 | | 0.287 |

**Supplementary Table S1.3.** Low-Beta power at different timepoints and brain regions with comparisons (* p<.05).

|  | Pre-treatment (Pre) | | Post-treatment (Post) | | Follow-up (FUP) | | Comparison | | | |
| --- | --- | --- | --- | --- | --- | --- | --- | --- | --- | --- |
|  | Mean (SD) | Median (IQR) | Mean (SD) | Median (IQR) | Mean (SD) | Median (IQR) | p_Pre-Post_ | | p_Post-FUP_ | p_Pre-FUP_ |
| Frontal | 0.713 (0.525) | 0.544 (0.305- 0.938) | 0.559 (0.342) | 0.403 (0.300- 0.749) | 0.593 (0.428) | 0.435 (0.301- 0.833) | 0.113 | 0.634 | | 0.141 |
| Temporal | 1.367 (1.009) | 1.043 (0.731- 1.508) | 1.202 (0.673) | 1.042 (0.701- 1.388) | 1.449 (1.114) | 0.953 (0.781- 1.665) | 0.751 | 0.042* | | 0.396 |
| Centro-parietal | 0.602 (0.544) | 0.510 (0.257- 0.704) | 0.492 (0.393) | 0.390 (0.284- 0.550) | 0.572 (0.540) | 0.414 (0.218- 0.701) | 0.134 | 0.252 | | 0.895 |
| Occipital | 1.712 (1.297) | 1.167 (0.865- 2.135) | 1.517 (1.231) | 0.987 (0.708- 2.297) | 1.885 (1.743) | 1.170 (0.767- 2.224) | 0.353 | 0.027* | | 0.312 |

**Supplementary Table S1.4.** High-Beta power at different timepoints and brain regions with comparisons.

|  | Pre-treatment (Pre) | | Post-treatment (Post) | | Follow-up (FUP) | | Comparison | | | |
| --- | --- | --- | --- | --- | --- | --- | --- | --- | --- | --- |
|  | Mean (SD) | Median (IQR) | Mean (SD) | Median (IQR) | Mean (SD) | Median (IQR) | p_Pre-Post_ | | p_Post-FUP_ | p_Pre-FUP_ |
| Frontal | 0.490 (0.441) | 0.353 (0.213-  0.534) | 0.390 (0.270) | 0.335 (0.151- 0.513) | 0.373 (0.258) | 0.294 (0.228- 0.489) | 0.312 | 1.000 | | 0.426 |
| Temporal | 0.679 (0.537) | 0.473 (0.389- 0.789) | 0.628 (0.427) | 0.468 (0.345- 0.771) | 0.695 (0.494) | 0.545 (0.430- 0.758) | 0.958 | 0.263 | | 0.474 |
| Centro-parietal | 0.285 (0.247) | 0.221 (0.150- 0.288) | 0.241 (0.171) | 0.176 (0.152- 0.281) | 0.279 (0.275) | 0.188 (0.138- 0.317) | 0.275 | 0.300 | | 0.937 |
| Occipital | 0.710 (0.636) | 0.469 (0.384- 0.889) | 0.604 (0.443) | 0.415 (0.310- 0.831) | 0.835 (0.939) | 0.537 (0.345- 1.133) | 0.230 | 0.156 | | 0.325 |

**Supplementary Table S2.1.** Theta power correlation coefficients with clinical outcomes (* p<.05; ** p<.01, *** p<.001).

|  | Pre - Post | | | | Post - FUP | | | | | Pre - FUP | | | | |
| --- | --- | --- | --- | --- | --- | --- | --- | --- | --- | --- | --- | --- | --- | --- |
|  | BSS | DASS (D) | DASS (A) | DASS (S) | BSS | DASS (D) | DASS (A) | DASS (S) | | | BSS | DASS (D) | DASS (A) | DASS (S) |
| Frontal | -0.027 | -0.177 | -0.076 | -0.205 | -0.056 | -0.313 | -0.055 | | -0.132 | | 0.033 | -0.046 | 0.024 | -0.113 |
| Temporal | -0.305 | -0.289 | -0.377 | -0.585  ** | -0.044 | 0.143 | -0.055 | | 0.099 | | 0.102 | -0.064 | -0.283 | -0.062 |
| Centro-parietal | -0.088 | -0.313 | -0.221 | -0.299 | 0.128 | -0.072 | -0.198 | | 0.039 | | 0.058 | -0.150 | -0.404  * | -0.139 |
| Occipital | -0.309 | -0.358 | -0.522  ** | -0.659  *** | 0.162 | 0.431* | 0.235 | | 0.526  ** | | 0.084 | -0.111 | -0.235 | 0.014 |

**Supplementary Table S2.2.** Alpha power correlation coefficients with clinical outcomes (* p<.05).

|  | Pre - Post | | | | Post - FUP | | | | | Pre - FUP | | | | |
| --- | --- | --- | --- | --- | --- | --- | --- | --- | --- | --- | --- | --- | --- | --- |
|  | BSS | DASS (D) | DASS (A) | DASS (S) | BSS | DASS (D) | DASS (A) | DASS (S) | | | BSS | DASS (D) | DASS (A) | DASS (S) |
| Frontal | 0.005 | -0.056 | 0.029 | -0.150 | 0.011 | -0.376 | -0.241 | | -0.372 | | -0.088 | -0.130 | 0.057 | -0.189 |
| Temporal | -0.143 | -0.159 | -0.231 | -0.358 | 0.100 | 0.213 | 0.278 | | 0.110 | | -0.109 | -0.196 | -0.455  * | -0.402  * |
| Centro-parietal | 0.089 | -0.115 | -0.053 | -0.120 | 0.312 | 0.055 | 0.150 | | 0.083 | | 0.008 | -0.092 | -0.349 | -0.248 |
| Occipital | -0.058 | -0.108 | -0.152 | -0.222 | 0.015 | 0.286 | 0.310 | | 0.229 | | -0.156 | -0.305 | -0.284 | -0.335 |

**Supplementary Table S2.3.** Low-beta power correlation coefficients with clinical outcomes (* p<.05; ** p<.01).

|  | Pre - Post | | | | Post - FUP | | | | | Pre - FUP | | | | |
| --- | --- | --- | --- | --- | --- | --- | --- | --- | --- | --- | --- | --- | --- | --- |
|  | BSS | DASS (D) | DASS (A) | DASS (S) | BSS | DASS (D) | DASS (A) | DASS (S) | | | BSS | DASS (D) | DASS (A) | DASS (S) |
| Frontal | 0.012 | -0.190 | 0.067 | -0.176 | -0.151 | -0.464  * | -0.224 | | -0.375 | | -0.057 | -0.203 | 0.032 | -0.288 |
| Temporal | -0.394 | -0.356 | -0.251 | -0.528  ** | -0.151 | -0.024 | 0.006 | | -0.078 | | -0.256 | -0.384 | -0.375 | -0.331 |
| Centro-parietal | -0.106 | -0.276 | -0.152 | -0.312 | 0.009 | -0.032 | 0.044 | | 0.053 | | -0.044 | -0.284 | -0.336 | -0.171 |
| Occipital | -0.248 | -0.249 | -0.234 | -0.506  ** | 0.044 | 0.379 | 0.169 | | 0.318 | | -0.149 | -0.421  * | -0.282 | -0.216 |

**Supplementary Table S2.4.** High-beta power correlation coefficients with clinical outcomes (* p<.05; ** p<.01).

|  | Pre - Post | | | | Post - FUP | | | | | Pre - FUP | | | | |
| --- | --- | --- | --- | --- | --- | --- | --- | --- | --- | --- | --- | --- | --- | --- |
|  | BSS | DASS (D) | DASS (A) | DASS (S) | BSS | DASS (D) | DASS (A) | DASS (S) | | | BSS | DASS (D) | DASS (A) | DASS (S) |
| Frontal | 0.097 | 0.030 | 0.315 | 0.055 | -0.388 | -0.571  ** | -0.211 | | -0.365 | | 0.075 | -0.070 | 0.215 | -0.181 |
| Temporal | -0.324 | -0.227 | -0.033 | -0.334 | -0.215 | -0.120 | 0.176 | | -0.073 | | -0.365 | -0.504  * | -0.443  * | -0.595  ** |
| Centro-parietal | -0.093 | -0.092 | 0.181 | -0.165 | -0.008 | -0.101 | 0.114 | | -0.029 | | -0.186 | -0.420  * | -0.181 | -0.204 |
| Occipital | -0.228 | -0.167 | -0.159 | -0.391 | -0.130 | 0.135 | -0.006 | | 0.051 | | -0.236 | -0.396 | -0.364 | -0.399  * |
